# The antitumour drug ABTL0812 impairs neuroblastoma growth through endoplasmic reticulum stress-mediated autophagy and apoptosis

**DOI:** 10.1038/s41419-020-02986-w

**Published:** 2020-09-17

**Authors:** Laia París-Coderch, Aroa Soriano, Carlos Jiménez, Tatiana Erazo, Pau Muñoz-Guardiola, Marc Masanas, Roberta Antonelli, Ariadna Boloix, José Alfón, Héctor Pérez-Montoyo, Marc Yeste-Velasco, Carles Domènech, Josep Roma, Josep Sánchez de Toledo, Lucas Moreno, José M. Lizcano, Soledad Gallego, Miguel F. Segura

**Affiliations:** 1 Translational Research in Child and Adolescent Cancer, Vall d’Hebron Institut de Recerca (VHIR), Barcelona, Spain; 2 Protein Kinases and Signal Transduction Laboratory, Institut de Neurociències and Departament de Bioquímica i Biologia Molecular, Universitat Autònoma de Barcelona, Bellaterra, Spain; 3 Ability Pharmaceuticals, SL, Avinguda Parc Tecnològic 3, Parc Tecnològic del Vallès, Cerdanyola, Spain; 4 Pediatric Oncology and Hematology Unit, Hospital Universitari Vall D’Hebron, Universitat Autònoma de Barcelona, Barcelona, Spain

**Keywords:** Drug development, Paediatric cancer

## Abstract

Neuroblastoma is the leading cause of cancer death in children aged 1 to 4 years. Particularly, five-year overall survival for high-risk neuroblastoma is below 50% with no curative options when refractory or relapsed. Most of current therapies target cell division and proliferation, thereby inducing DNA damage and programmed cell death. However, aggressive tumours often present alterations of these processes and are resistant to therapy. Therefore, exploring alternative pathways to induce tumour cell death will provide new therapeutic opportunities for these patients. In this study we aimed at testing the therapeutic potential of ABTL0812, a novel anticancer drug that induces cytotoxic autophagy to eliminate cancer cells, which is currently in phase II clinical trials of adult tumours. Here, we show that ABTL0812 impaired the viability of clinical representative neuroblastoma cell lines regardless of genetic alterations associated to bad prognosis and resistance to therapy. Oral administration of ABTL0812 to mice bearing neuroblastoma xenografts impaired tumour growth. Furthermore, our findings revealed that, in neuroblastoma, ABTL0812 induced cancer cell death via induction of endoplasmic reticulum stress, activation of the unfolded protein response, autophagy and apoptosis. Remarkably, ABTL0812 potentiated the antitumour activity of chemotherapies and differentiating agents such as irinotecan and 13*-cis*-retinoic acid. In conclusion, ABTL0812 distinctive mechanism of action makes it standout to be used alone or in combination in high-risk neuroblastoma patients.

## Introduction

Neuroblastoma is an embryonic neuroendocrine tumour of the sympathetic nervous system, representing 8–10% of all diagnosed childhood cancers in western countries and ~15% of all cancer-related deaths in children. In Europe, the estimated annual incidence is ~1000 new cases per year, of which 60% are classified as high-risk, with a 5-year overall survival below 50%^1–3^. Furthermore, a significant proportion of patients will suffer major treatment-associated long-term side effects and life-long disabilities^2^.

Autophagy is an evolutionary-conserved catabolic process that recycles unnecessary or dysfunctional cellular components^4^. In cancer, autophagy has been proposed to suppress malignant transformation by controlling cell growth, degradation of oncogenic/damaged proteins, preservation of genetic/genomic stability and induction of autophagic cell death among other mechanisms. However, in established tumours, autophagy may also promote tumour progression and resistance to therapy through conferring resistance to hypoxia and nutrient deprivation, induction of dormancy; disabling immunosurveillance or facilitating EMT transition and dissemination [reviewed in refs. ^5,6^]. In neuroblastoma, several compounds have already shown to exert antitumour activity through the activation of autophagy^7–10^.

The novel compound ABTL0812 is the sodium salt formulation derivative from 2-hydroxy-linoleic acid, an essential polyunsaturated ω6 fatty acid made up of eighteen carbons (chemical name: sodium 2-hydroxylinoleate). ABTL0812 triggers cytotoxic autophagy through inhibition of the AKT/mTOR pathway^11^ and through the sustained induction of endoplasmic reticulum (ER) stress and activation of the unfolded protein response (UPR)^12^. Of note, these two pathways have been shown to modulate the expression or stability of MYCN, a key undruggable genetic driver in neuroblastoma^13,14^. Therefore, we decided to test the therapeutic potential of ABTL0812 in neuroblastoma.

ABTL0812 successfully completed a Phase I/Ib trial in patients with advanced solid tumours (NCT02201823), showing a good safety and tolerability profile. Moreover, first signs of efficacy were observed^15^. These satisfactory results led to the ongoing Phase I/II trials in patients with advanced endometrial cancer and squamous non-small cell lung cancer, where ABTL0812 is given at first line in combination with chemotherapy (NCT03366480).

Here, we present evidence that ABTL0812 impairs neuroblastoma tumour growth, both in vitro and in vivo through ER stress and UPR activation, leading to cytotoxic autophagy and apoptosis. Furthermore, our results suggest that in neuroblastoma ABTL0812 enhances the activity of standard therapies, such as irinotecan and the differentiating agent 13*-cis*-retinoic acid (13-cRA), thus providing a new therapeutic option for high-risk neuroblastoma patients.

## Materials and methods

### Cell lines

SK-N-AS, SH-SY5Y, and IMR-32 cell lines were purchased from American Type Culture Collection (ATCC, Manassas, VA, USA), CHLA-90 cell line was acquired from the Children’s Oncology Group Cell Culture and Xenograft Repository (Lubbock, TX, USA), and SK-N-BE(2) and LA1-5s were purchased from Public Health England Culture Collections (Salisbury, UK). Neuroblastoma cell lines were cultured and maintained in Iscove’s modified Dulbecco’s medium (Thermo Fisher Scientific, Waltham, MA, USA) supplemented with 10% heat-inactivated foetal bovine serum (FBS, South America Premium, Bio-west), 1% of insulin–transferrin–selenium Supplement (Thermo Fisher Scientific), 100 U/mL penicillin, 100 μg/mL streptomycin (Thermo Fisher Scientific) and 5 μg/mL plasmocin (InvivoGen, San Diego, CA, USA). All cultures were maintained at 37 °C in a saturated atmosphere of 5% CO_2_. All cell lines were periodically tested for mycoplasma contamination.

### Reagents

ABTL0812 was provided by Ability Pharmaceuticals (Cerdanyola del Vallès, Spain) and cis-Diammineplatinum(II) dichlorideDNA (Cisplatin) was purchased from Sigma-Aldrich (St. Louis, MO, USA). Therapeutic agents doxorubicin hydrochloride, irinotecan (SN-38), topotecan, cyclophosphamide monohydrate and 13-*cis*-retinoic acid (Isotretinoin) were purchased from Selleckchem (Houston, TX, USA). Autophagy inhibitors E64d (Cysteine-catepsines inhibitor), Pepstatin A (Aspartic-proteases inhibitor) and the pan-caspase inhibitor QVD-OPh were purchased from Sigma-Aldrich.

### Cell proliferation assays

Neuroblastoma cells were seeded in 96-well plates at 6–16 × 10^3^ cells/well (*n* = 6/condition) and 24 h later treated with the designated drugs diluted in culture medium supplemented with 0.5% FBS. At the indicated times, cells were fixed with 1% glutaraldehyde (Sigma-Aldrich) and stained with 0.5% crystal violet (Sigma-Aldrich). Crystals were dissolved in 15% acetic acid (Thermo Fisher Scientific) and absorbance was measured at 590 nm using an Epoch Microplate Spectrophotometer (Biotek, Winooski, Vermont, USA).

### Mouse xenografts

All animal procedures were approved by the Ethics Committee for Animal Experimentation of Vall Hebron Research Institute (ref-12/14 CEEA).

SH-SY5Y cells were injected in the flank of 6-week-old female NMRI-Foxn1^nu/nu^ immunodeficient mice (5 × 10^6^ cells/flank) in 300 μl of PBS and Matrigel^TM^ (Corning, NY, USA) in proportion 1:1. Sample size was calculated using the G*Power software version 3.19.6 (University of Kiel, Germany). Power was set to detect differences with *P* value < 0.05 and a 50% difference in tumour growth. Once all mice developed measurable tumours (~70 mm^3^), mice were allocated in three different groups by block randomisation. The three groups were vehicle (5% glycerol in water; *n* = 10), 120 mg/kg ABTL0812 (*n* = 10) or 2 mg/kg cisplatin (*n* = 10). ABTL0812 and vehicle were orally administered 7 days/week. Cisplatin was diluted in PBS and administered i.p. 2 days/week. Tumour growth and mice weight was measured with no blinding using a digital calliper 2–3 times per week. When primary tumours reached 1500 mm^3^, mice were euthanized and tumours excised and weighted. Only those tumours confirmed by haematoxylin and eosin staining were included in the analyses.

### Immunofluorescence

Cells were seeded in 24-well plates (8 × 10^4^ cells/well for SK-N-BE(2); 6 × 10^4^ cells/well for LA1-5s), on slide covers coated with 40 μg/ml poly-L-lysine (Sigma-Aldrich). After 24 h, cells were treated with the indicated treatments for 12 h. Cells were fixed with 4% paraformaldehyde (Sigma-Aldrich), permeabilized with 0.02% saponin (Sigma-Aldrich) and blocked for 1 h with 0.01% saponin, 10 mM glycine and 5% BSA (bovine serum albumin; Sigma-Aldrich). Next, cells were incubated overnight with the LC3 primary antibody diluted in 0.01% saponin and 1% BSA in a wet chamber at 4 °C, and then incubated for 1 h with the secondary antibody. Nuclei were stained with Hoechst 33342 (Sigma-Aldrich) for 5 min, rinsed and mounted on a slide with a drop of Fluorsave mounting medium (Calbiochem, Darmstadt, Germany). Images were captured using a fluorescent microscope (Nikon Eclipse 90i, Nikon, Vienna, Austria).

### Western blot

Proteins were extracted using RIPA buffer (Thermo Fisher Scientific) supplemented with 1× EDTA-free complete protease inhibitor cocktail (Roche, Basel, Switzerland) and the phosphatase inhibitors sodium fluoride and sodium orthovanadate (Sigma-Aldrich). Protein concentration was determined using Lowry DC protein assay (Bio-Rad, Hercules, CA, USA). Cell lysates (25–30 μg of protein) were resolved on NuPAGE^TM^ 4–12% Bis-Tris gels (Thermo Fisher Scientific) and transferred into PVDF membranes. Membranes were blocked for 1 h with 5% non-fat milk or 5% BSA diluted in Tris-buffered saline 0.1% Tween and probed with the indicated primary antibodies (see Supplementary Table 1) overnight at 4 °C. Membranes were incubated with secondary antibodies for 1 h and developed with chemiluminescent horseradish peroxidase substrate Immobilon Western (Millipore, Darmstadt, Germany). Quantifications of western blots were performed with Image J (National Institutes of Health, Bethesda, MD, USA).

### Cell death assays

Cells were seeded in 24-well plates and treated 24 h later with ABTL0812 or cisplatin. At the end of treatment, cells were stained with 0.05 μg/ml Hoechst 33342 (Sigma-Aldrich) and 2.5 μM propidium iodide (PI, Thermo), and incubated for 15 min protected from light. Images were captured using a fluorescent microscope (Nikon Eclipse 90i, Nikon). Cells with condensed or fragmented nuclei were considered as dead cells and those with uniformly stained chromatin as healthy. Scoring was evaluated by blinded investigators. A minimum of 100 cells were counted from four representative areas of each well.

### Statistical analysis

Unless otherwise stated, graphs represent the average of three independent experiments ± SEM. Statistical significance was determined by unpaired two-tailed Student’s *t*-test or ANOVA Tukey’s test (GraphPad Prism Software, USA).

## Results

### ABTL0812 impairs neuroblastoma cell growth in vitro and tumour growth in vivo

**Fig. 1:**
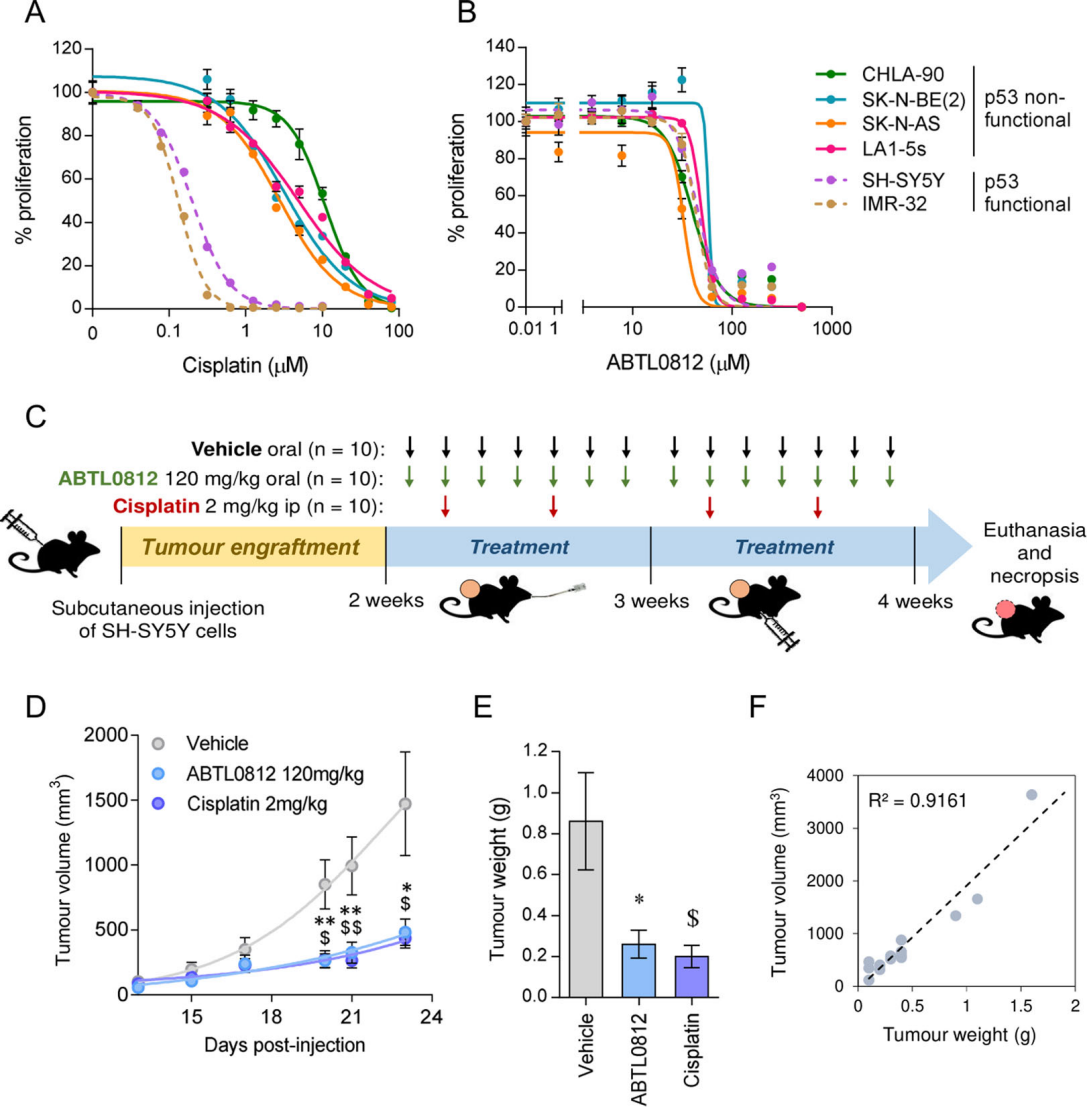
ABTL0812 reduces neuroblastoma cell lines proliferation in vitro and tumour growth in vivo. **a, b** Dose-response curves for cisplatin (**a**) and ABTL0812 (**b**) in six neuroblastoma cell lines. Cells were treated with ABTL0812 or cisplatin for 72 h at the indicated concentrations, then fixed with 1% glutaraldehyde and stained with crystal violet. **c** Schematic representation of the in vivo experiment using a neuroblastoma xenograft model. **d** Tumour volume of mice treated with vehicle, ABTL0812 or cisplatin measured at 23 days (*n* = 10/group). **e** Average weight of resected tumours. **f** Correlation between tumour weight and tumour volume. Data is presented as mean ± SEM. *p ≤ 0.05, **p ≤ 0.01 ABTL0812 vs. vehicle; ^$^*p* ≤ 0.05, ^$$^*p* ≤ 0.01 cisplatin vs. vehicle.

A panel of neuroblastoma cell lines with clinically relevant molecular alterations (Supplementary Table 2) was selected to analyse their sensitivity to ABTL0812 or cisplatin, one of the standards-of-care DNA-damaging agents used in the induction phase of high-risk neuroblastoma treatment^16^. While cisplatin was much more effective in neuroblastoma cell lines with functional p53, i.e., SH-SY5Y and IMR-32 (Fig. 1a), ABTL0812 impaired the growth of all tested cell lines, regardless of p53 functional status or genomic alterations such as *MYCN* amplification (Fig. 1b). IC_50_ values for ABTL0812 were in the similar range (i.e., 30–60 µM) among all tested cell lines. However, IC_50_ values for cisplatin were at least 10 times lower in the p53 wild type cell lines compared to p53 mutant cell lines (Supplementary Table 3).

To identify potential adverse effects resulting from exposure to ABTL0812 in children, an Ames test^17^ was performed, which showed no mutagenic effect (Supplementary Fig. 1A, B). Furthermore, in neuroblastoma cells, ABTL0812 induced little or no phosphorylation of H2AX at serine 139 (a marker of DNA double strand breaks^18^), while p-H2AX was strongly induced by cisplatin (Supplementary Fig. 1C).

The impact of ABTL0812 treatment on neuroblastoma cells was further evaluated in vivo using subcutaneous xenografts (Fig. 1c). While tumours of vehicle-treated mice showed a rapid growth, those treated with ABTL0812 or cisplatin grew at a much slower pace (Fig. 1d). These effects were confirmed by analysis of tumour weight at the end of the experiment (Fig. 1e) and validated by a strong correlation between tumour weight and volume (Fig. 1f). Mice weight measurements showed a slight reduction in body weight in ABTL0812 treated group (~5%, Supplementary Fig. 2A). We also did a complete blood cell count test (CBC) from five to twelve mice from each treatment group. The haematological analysis showed that ABTL0812 had no impact on haematocrit, haemoglobin and red blood cell counts, neither in liver and kidney damage indicators AST, ALT and urea concentration (Supplementary Fig. 2B).

Collectively, these data show that ABTL0812 has a similar antitumour potential to cisplatin. Conversely, it does cause neither DNA strand breaks nor mutations.

### ABTL0812 induces cell death in neuroblastoma cell lines

**Fig. 2:**
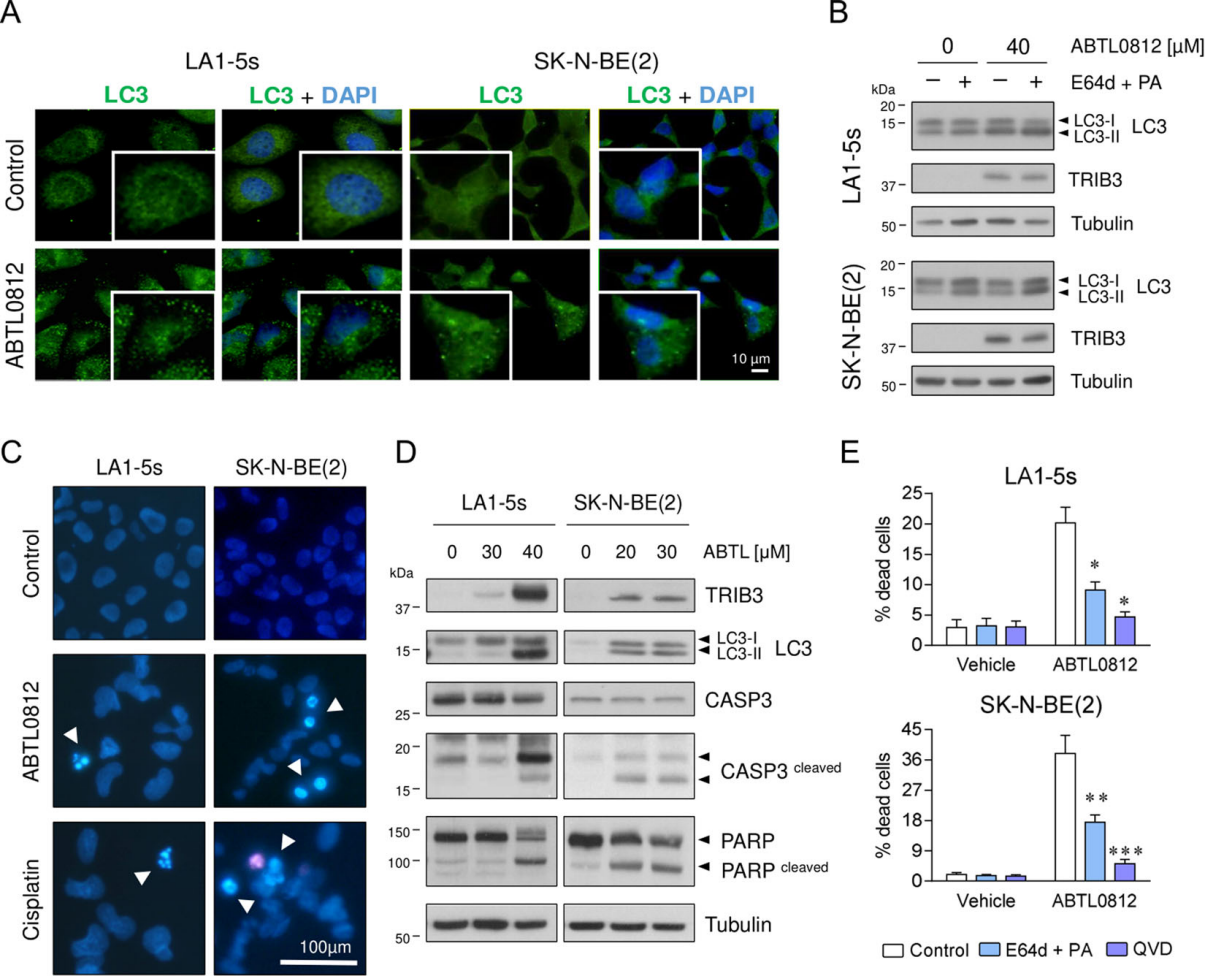
ABTL0812 induces autophagy and apoptosis in neuroblastoma cells. **a** Immunostaining of ABTL0812-induced autophagosome formation. Neuroblastoma cells were treated with 20 µM (SK-N-BE(2)) or 40 µM (LA1-5s) ABTL0812 for 12 h and then fixed and stained with anti-LC3 (green) and DAPI (blue). Punctuated patterns shows LC3-II recruited to the autophagosomes. **b** Western blot analysis of ABTL0812-induced autophagic flux. Cells were pre-treated 2 h with vehicle or 10 µM E64d and 10 µg/ml pepstatin A (PA). Then, 40 µM (LA1-5s) or 30 µM (SK-N-BE(2)) ABTL0812 was added for 6 h in the presence or absence of lysosomal inhibitors. Anti-TRIB3 was used as a control for ABTL0812 response. **c** Representative images of nuclear morphology assessment at 48 h post-treatment with ABTL0812 (30 µM for LA1-5s, 20 µM for SK-N-BE(2)) or vehicle. Arrowheads point towards condensed or fragmented nuclei. Cisplatin (25 µM) was used as an apoptosis positive control. **d** Western blot analysis of ABTL0812-induced apoptosis 72 h post-treatment. **e** Autophagy and apoptosis inhibitors reduce ABTL0812-induced cell death. LA1-5s and SK-N-BE(2) cells were pre-treated 2 h with vehicle (ethanol) or 10 μM E64d and 10 μg/ml PA or 20 μM QVD. 30 μM ABTL0812 for LA1-5s or 20 μM for SK-N-BE(2) were added in the presence or absence of autophagy and apoptosis inhibitors. Cell death was quantified 48 h post-treatment by scoring four representative fields of each condition (*n* = 3). Data is presented as the average of three independent experiments ± SEM. *p ≤ 0.05, **p ≤ 0.01, ***p ≤ 0.001 compared to ABTL0812 in the absence of inhibitors.

ABTL0812 is known to impair tumour growth by activation of autophagy-mediated cell death in multiple adult cancers^11,12,19,20^. During autophagy, the cytosolic soluble protein LC3 is converted to LC3-II, which is bound to autophagosomal membranes. Thus, the diffuse ubiquitous LC3 distribution pattern is converted into a punctuate/dot LC3-II pattern, a process that can be tracked by immunofluorescence as a hallmark of autophagy. When chemoresistant neuroblastoma cells were treated with ABTL0812, a remarkable change in LC3 distribution was observed, showing an increase of punctuate/dot LC3-II in both cell lines (Fig. 2a).

Cytotoxic autophagy only occurs when the autophagic flux is completed and autophagosomes fuse with lysosomes to be degraded, leading to the concomitant loss of LC3-II. Since pre-incubation of cells with lysosomal degradation inhibitors (E64d and pepstatin A) resulted in increased levels of LC3-II, it can be assumed that ABTL0812 induced dynamic autophagic flux (Fig. 2b). Interestingly, at later time points, Hoechst staining of ABTL0812-treated neuroblastoma cell lines showed the typical morphological features of apoptosis: chromatin condensation, nuclear fragmentation and formation of apoptotic bodies (Fig. 2c), thereby suggesting that ABTL0812 treatment also induced apoptosis in neuroblastoma cells. Indeed, cleavage of caspase-3 and its substrate PARP was also observed upon ABTL0812 treatment (Fig. 2d). Moreover, pre-treatment with the lysosomal protease inhibitors E64d/pepstatin A or with the pan-caspase inhibitor QVD-OPh (QVD) resulted in a significant reduction of ABTL0812-induced cell death (Fig. 2e). Collectively, these results suggest that ABTL0812 treatment triggers autophagy and apoptosis in neuroblastoma cell lines.

### ABTL0812 induces endoplasmic reticulum stress and the unfolded protein response

**Fig. 3:**
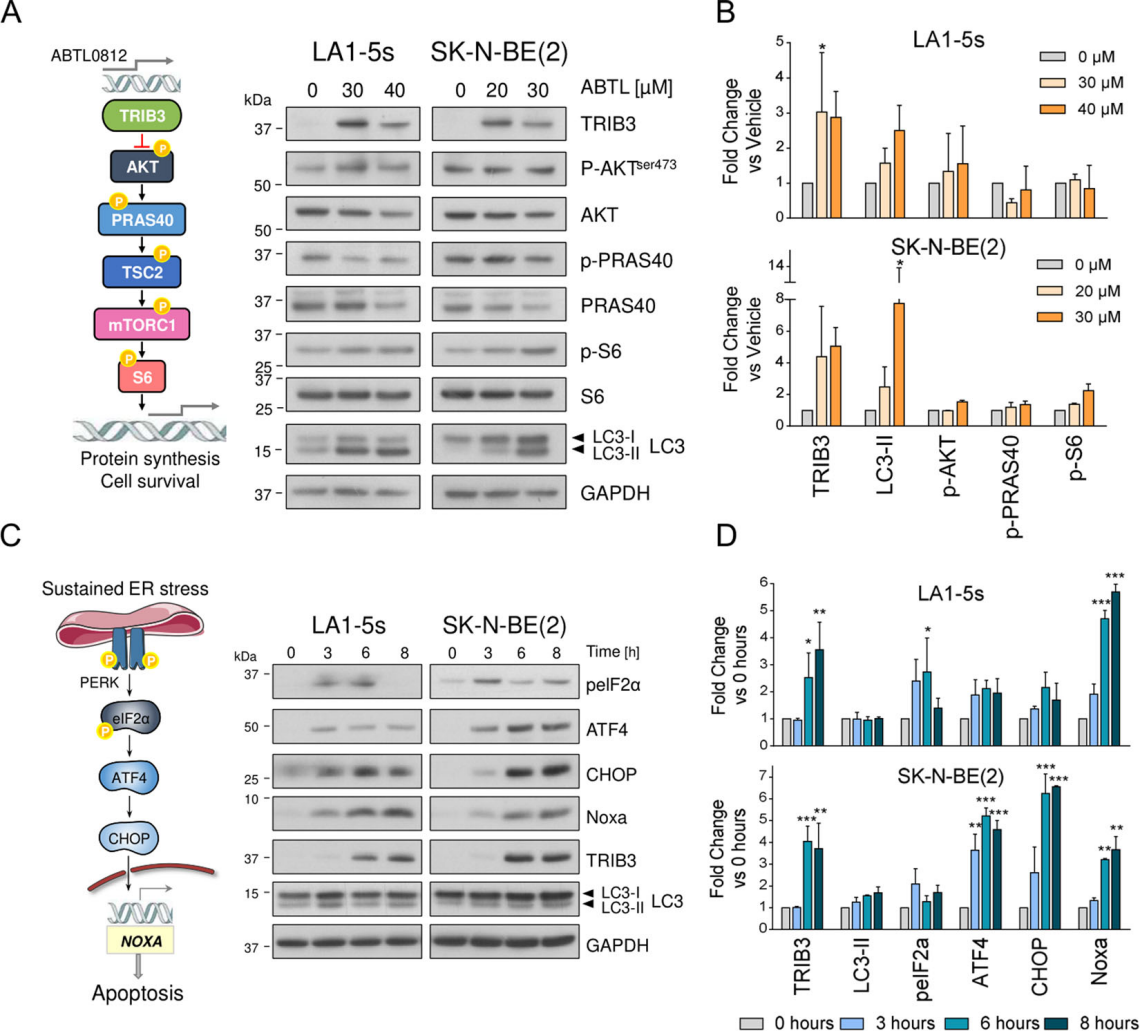
ABTL0812 triggers the UPR-PERK signalling pathway. **a** Left, representative scheme of the AKT/mTORC1 pathway. Right, western blot of the components of the pathway in LA1-5s and SK-N-BE(2) cell lines treated with the indicated ABTL0812 concentrations for 24 h. **b** Western blot quantification. Graph represents the mean of two out of three independent experiments. Band intensity was normalised to GAPDH and vehicle-treated cells. *p ≤ 0.05, compared to vehicle. **c** Left, representative scheme of the UPR PERK pathway. Right, western blot of the components of the pathway in LA1-5s and SK-N-BE(2) cell lines treated with the indicated ABTL0812 concentrations for 0, 3, 6 and 8 h. **d** Graph represents the mean quantification of two out of three independent experiments. Band intensity was normalised to GAPDH and time 0 h. *p ≤ 0.05, **p ≤ 0.01, ***p ≤ 0.001 compared to time = 0.

To shed light on the molecular pathways behind ABTL0812-induced cell death, we investigated the first proposed mechanism of action for ABTL0812, the inhibition of the AKT/mTORC1 axis by upregulation of TRIB3 pseudokinase^11^. Although treatment of neuroblastoma cells with ABTL0812 resulted in increased TRIB3 levels and bi-lipidation of LC3, the levels of AKT phosphorylation or its downstream targets PRAS40 and S6 were not modified at the time and doses analysed (Fig. 3a, b).

Recently, ABTL0812 has also been shown to modulate cytotoxic autophagy through induction of ER stress and the UPR^12^. We monitored the impact of ABTL0812 on the PERK signalling pathway, a branch of UPR closely related to ER stress-induced apoptosis^21–24^. ABTL0812 treatment resulted in increased phosphorylation of PERK substrate eIF2α, as well as in increased protein levels of the downstream effectors ATF4 and the C/EBP homologous protein (CHOP) (Fig. 3c, d). Although the exact mechanism of how these effectors trigger apoptotic cell death is not completely understood, they usually do through the activation of pro-apoptotic members of the Bcl-2 family such as Bim, NOXA or Puma^25^. Concurring with these observations, a remarkable upregulation of NOXA was seen in both neuroblastoma cell lines analysed upon ABTL0812 treatment (Fig. 3c, d).

In summary, these data support the hypothesis that ABTL0812 induces neuroblastoma cell death through induction of ER stress and the PERK branch of the UPR pathway.

### ABTL0812 potentiates the antitumoural effect of chemotherapy and differentiating agents used for the management of high risk-neuroblastoma

**Fig. 4:**
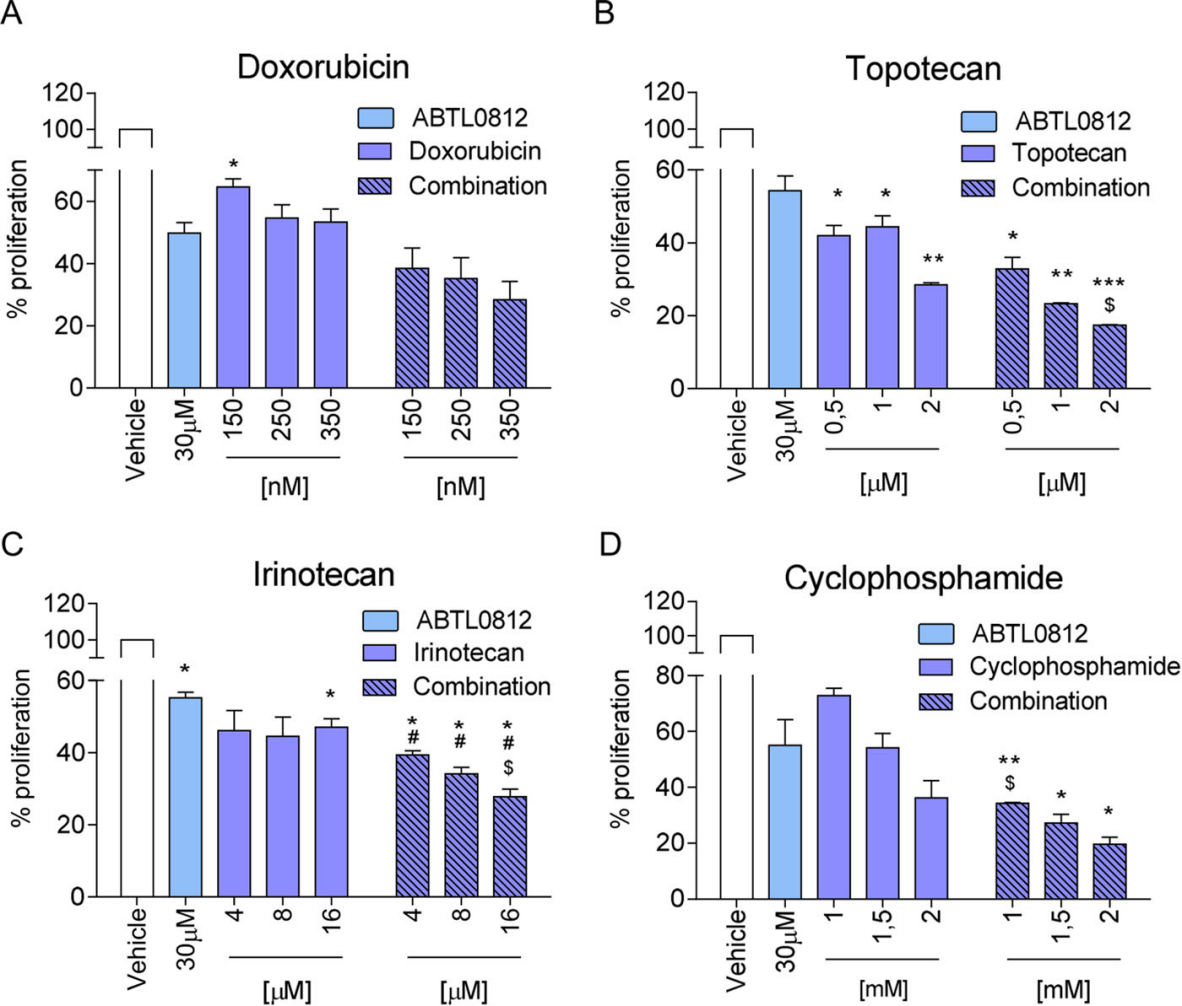
ABTL0812 potentiates the antitumoural effect of high-risk neuroblastoma treatments. LA1-5s cells were treated with vehicle (ethanol), 30 µM ABTL0812 and the indicated doses of doxorubicin (**a**), topotecan (**b**), irinotecan (**c**) and cyclophosphamide (**d**) as a single agent (plain bars) or in combination with 30 µM ABTL0812 (striped bars). After 72 h of treatment, cells were fixed with 1% glutaraldehyde and proliferation was assessed by crystal violet staining. Data is presented as the average of three independent experiments ± SEM. *, #, $ mean *p* ≤ 0.05, **p ≤ 0.01. “*” indicates comparison vs. vehicle; “#” compares vs. ABTL0812 as a single agent; and “$” compares vs. the indicated drug as single agent.

Currently, high-risk neuroblastoma is treated with regimens of combined chemotherapies. Hence we aimed to combine ABTL0812 with the most common drugs used for patients with high-risk neuroblastoma, such as doxorubicin, irinotecan, topotecan and cyclophosphamide. Three different doses of each compound, alone or in combination with 30 µM ABTL0812 were tested (Fig. 4a-d). While a positive trend was observed for all compounds, the strongest antitumoural effect was seen when ABTL0812 was combined with irinotecan. At the three tested concentrations, the addition of irinotecan increased the cytotoxic effect of ABTL0812 (Fig. 4c).

**Fig. 5:**
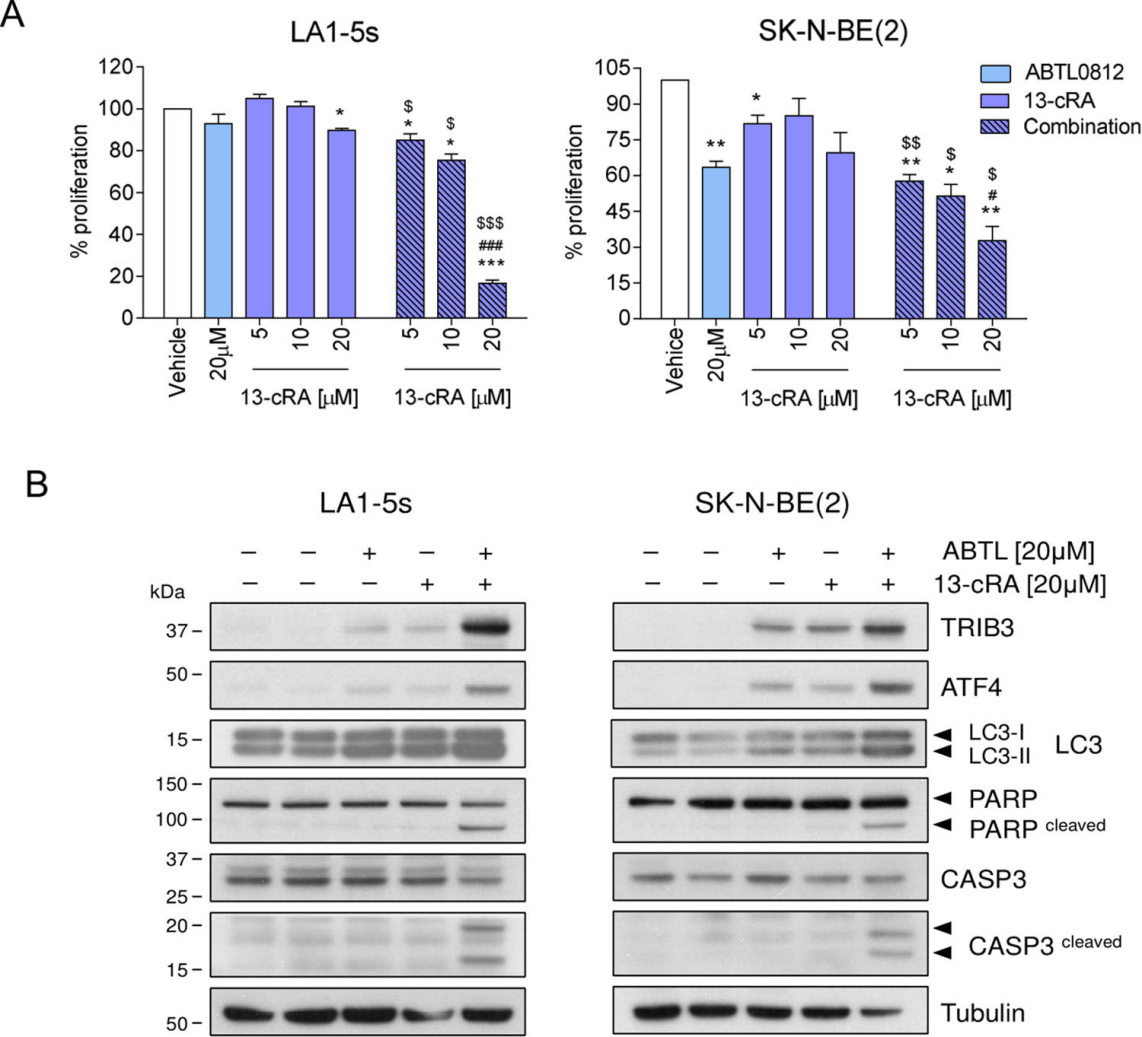
ABTL0812 potentiates the antitumoural effect of 13-*cis*-retinoic acid. LA1-5s and SK-N-BE(2) cells were treated with vehicle (ethanol), 20 µM ABTL0812 or the indicated doses of 13-cRA, alone or in combination with ABTL0812. **a** Cell proliferation assay. Neuroblastoma cells were treated for 24 h and then fixed with 1% glutaraldehyde and stained with crystal violet. Graph represents the average of three independent experiments ± SEM. Plain bars represent single agent treatments and striped bars combined treatments. *, #, ^$^*p* ≤ 0.05, **, ^$$^*p* ≤ 0.01, ***, ^###^*p* ≤ 0.001. “*” indicates comparison vs. vehicle; “#” compares vs. ABTL0812 as a single agent; and “$” compares vs. 13-cRA as single agent. **b** Western blot of the indicated proteins after treatment with both vehicles, 20 µM of ABTL0812, 20 µM of 13-cRA or the combination, for 24 h.

Finally, we investigated the effect of ABTL0812 combined with biologic agents used for the treatment of minimal residual disease (MRD) in neuroblastoma, such as 13-*cis*-retinoic acid (13-cRA). Cell proliferation assays were performed in LA1-5s and SK-N-BE(2) neuroblastoma cell lines treated simultaneously with ABTL0812 and three doses of 13-cRA. Administration of low 13-cRA doses as single agent had little effect on neuroblastoma cell proliferation at 24 h post-treatment; however, the combination of 13-cRA with ABTL0812 induced a strong reduction in the viability of both neuroblastoma cell lines (Fig. 5a). Interestingly, both drugs, ABTL0812 and 13-cRA, caused an upregulation of ABTL0812 effectors such as TRIB3, ATF4 and bi-lipidation of LC3, which were further enhanced when both drugs were combined. Concurring with this evidence, the apoptotic response was observed only when 13-cRA and ABTL0812 were added together. In both cell lines, the combined treatment induced the cleavage of caspase-3 and its substrate PARP-1, confirming activation of apoptosis (Fig. 5b).

In conclusion, these results suggest that ABTL0812 potentiates the antitumoural effect of high-risk neuroblastoma chemotherapy drugs and differentiating agents used for the treatment of minimal residual disease.

## Discussion

ABTL0812 is a first-in-class chemically modified polyunsaturated fatty acid which has demonstrated antitumour activity in several in vitro and in vivo models of adult cancers^11,19,20^. Our results show that ABTL0812 impairs neuroblastoma viability regardless of poor prognosis markers, such as *MYCN* amplification and p53 mutations. Unlike what it has been described for lung, pancreatic and endometrial cancer cells (6,10,11), ABTL0812 does not reduce phosphorylation of AKT or mTORC1 substrates, despite inducing the upregulation of TRIB3 and LC3-II levels. Thus, autophagy-mediated cell death in neuroblastoma is independent of the AKT/mTOR axis. In addition, we observed that neuroblastoma cells died by apoptosis, in accordance to what was observed for endometrial cancer models (10). Concurring with recent findings^12^, our results suggest that ABTL0812 induces prolonged ER stress and activation of the PERK/ATF4/CHOP/NOXA UPR branch. This novel mechanism of action would help to overcome some of the resistance mechanisms present in high-risk neuroblastoma tumours, such as defective p53 signalling and caspase 8 silencing^26–28^.

Cumulative preclinical data and the first-in-human phase I/Ib clinical trial of ABTL0812 as a single therapy (NCT02201823) showed high safety and tolerability. In fact, the maximum tolerated dose was not achieved because no dose‐limiting toxicities were identified^11,15,19,20^. Accordingly, our results confirmed that ABTL0812 is not mutagenic and does not induce DNA damage. Nonetheless, the in vivo efficacy of ABTL0812 is similar to cisplatin, a DNA-damaging chemotherapy drug used for neuroblastoma treatment. These results promote ABTL0812 as a potential therapeutic option for neuroblastoma patients with a higher safety profile than current treatments. This preclinical data supported the ABTL0812 Orphan Drug Designation (ODD) by the FDA (15‐4893) and EMA (EU/3/15/1485) agencies for neuroblastoma.

ABTL0812 has also shown synergistic effect in adult tumours when combined with standard chemotherapies^19,20^. Therefore, we analysed the potential benefit of combining ABTL0812 with chemotherapies used to treat high-risk neuroblastoma, either in first or second line, such as irinotecan, topotecan, doxorubicin and cyclophosphamide. Although our results suggest that ABTL0812 enhanced the therapeutic effect of these drugs, the potentiation effect was modest. However, we did see a remarkable effect when we combined ABTL0812 with 13c-RA, one of the biological agents used for the treatment of minimal residual disease in neuroblastoma^29,30^. Our molecular assays showed that low doses of 13-cRA were activating the PERK/ATF4/CHOP axis and autophagy, but not cell death. It has been reported that cell death only occurs through the UPR when the ER stress stimuli is intense and prolonged (reviewed in ref. ^31^). Consequently, when ABTL0812 was combined with 13c-RA, the ER stress was sufficiently enhanced reaching the threshold in which the survival/death scale is tipped towards the induction of apoptosis. In fact, the apoptotic phenotype is already observed at 24 h when ABTL0812 is combined with 13-cRA, whereas the apoptotic hallmarks are only visible 48 or 72 h post-treatment when ABTL0812 is used as a single agent.

In summary, our results strengthen the notion that prolonged and intense ER stress and UPR induction by ABTL0812 offers a new, safe and effective anti-cancer therapeutic strategy for patients with high-risk neuroblastoma.

## Supplementary information

Supplementary Figure 1

Supplementary Figure 2

Supplementary data File
