# Supplementary figures and images for: The antitumour drug ABTL0812 impairs neuroblastoma growth through endoplasmic reticulum stress-mediated autophagy and apoptosis
Source: Cell Death Dis. 2020 Sep 17;11(9):773. doi: 10.1038/s41419-020-02986-w (PMC7498451; doi:10.1038/s41419-020-02986-w)

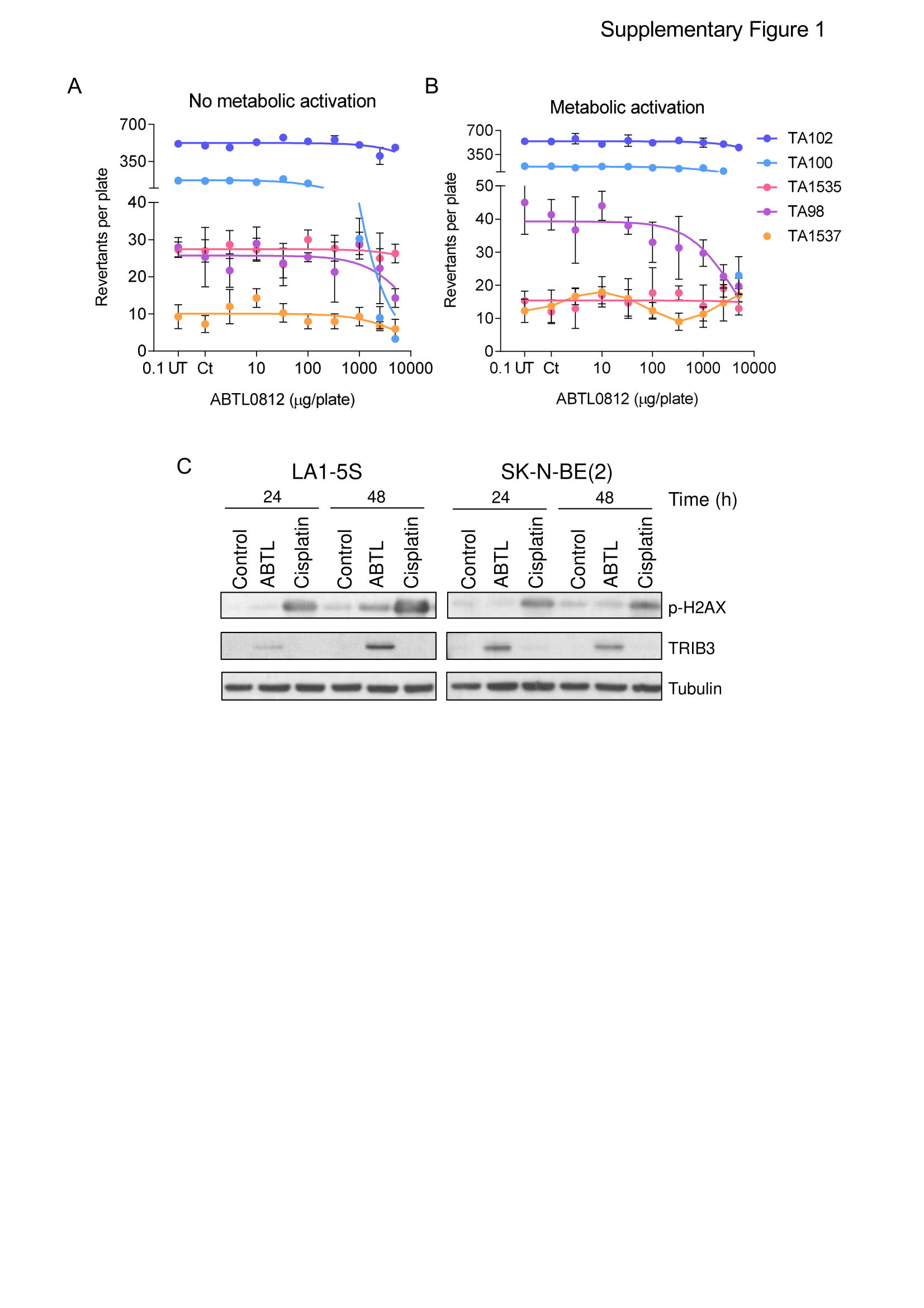

Supplement: Supplementary file 1 — Supplementary Figure 1 [file 41419_2020_2986_MOESM1_ESM.tif]

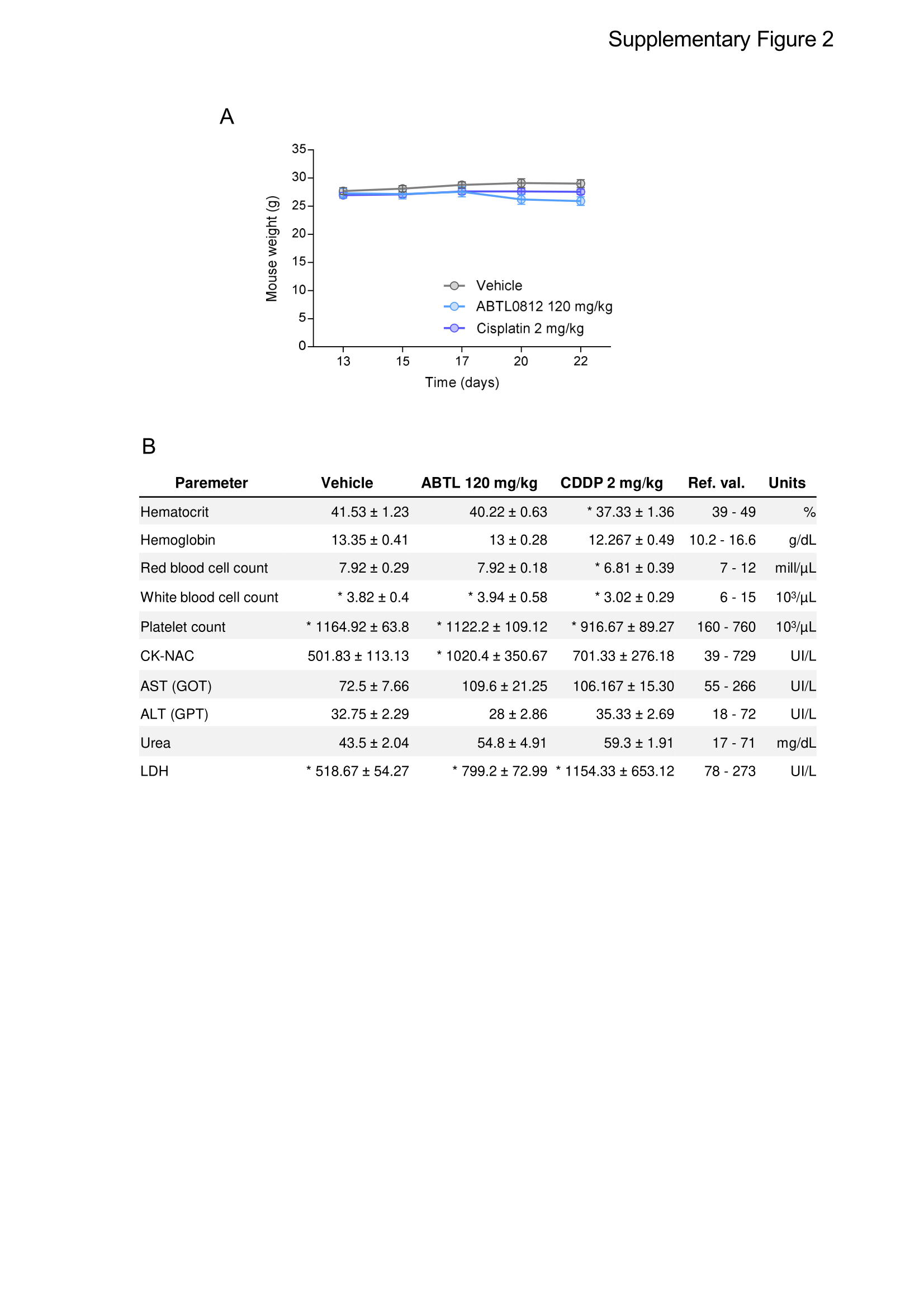

Supplement: Supplementary file 2 — Supplementary Figure 2 [file 41419_2020_2986_MOESM2_ESM.tif]
