# Supplementary material for: The antitumour drug ABTL0812 impairs neuroblastoma growth through endoplasmic reticulum stress-mediated autophagy and apoptosis
Source: Cell Death Dis. 2020 Sep 17;11(9):773. doi: 10.1038/s41419-020-02986-w (PMC7498451; doi:10.1038/s41419-020-02986-w)
Supplement: Supplementary file 3 — Supplementary data File [file 41419_2020_2986_MOESM3_ESM.docx]

**Supplementary Material**

**Chemical bacterial reverse mutation assay (Ames test)**

The auxotrophic mutant Salmonella typhimurium strains TA98, TA100, TA102, TA1535 and TA1537 (Trinova Biochem), unable to synthesize histidine from the ingredients of culture media, were spread on agar plates suspended in growth medium complemented with different ABTL0812 concentrations (3-5000 μg/plate), with or without the microsomal fraction S9 (concentration 38mg/ml) and with small amounts of histidine. The spread plates were incubated forty-eight hours at 37ºC before proceeding to count the number of colonies in each plate. Determination of the number of revertant colonies induced by ABTL0812 with or without S9 metabolic activation is considered proportional to its mutagenicity.

**Supplementary table 1:** Antibodies

| **Primary antibodies** | | | |
| --- | --- | --- | --- |
| ***Antibody*** | ***Dilution*** | ***Supplier*** | ***Reference*** |
| AKT | 1:5000 | CST | #9272 |
| ATF4 | 1:2000 | CST | #11815 |
| Caspase-3 | 1:3000 | CST | #9662 |
| Caspase-3 cleaved | 1:750 | CST | #9664 |
| CHOP | 1:1000 | CST | #2895 |
| eIF2α | 1:1000 | CST | #5324 |
| GAPDH | 1:10000 | SCBT | sc-32233 |
| LC3 | 1:10000 | Abcam | ab48394 |
| NOXA | 1:300 | Merck | #114C307 |
| p-AKT^ser473^ | 1:500 | CST | #9271 |
| p-eIF2α | 1:2000 | CST | #3398 |
| p-H2AX | 1:1000 | Merck | #05-636 |
| p-PRAS40 | 1:2000 | CST | #2997 |
| p-S6 | 1:10000 | CST | #4858 |
| PARP | 1:5000 | CST | #9542 |
| PRAS40 | 1:1000 | MRC PPU | S115B |
| TRIB3 | 1:3000 | Abcam | ab50516 |
| Tubulin | 1:20000 | Sigma-Aldrich | T5168 |
| S6 | 1:5000 | CST | #2217 |
| **Secondary antibodies** |  |  |  |
| Anti-Rabbit IgG-Peroxidase | 1:10000 | Sigma-Aldrich | #A0545 |
| Anti-Mouse IgG-Peroxidase | 1:10000 | Sigma-Aldrich | #A9044 |
| Anti-Sheep IgG-Peroxidase | 1:10000 | Sigma-Aldrich | #A3415 |
| Anti-Rabbit IgG Alexa Fluor^®^ 594 | 1:10000 | Thermo-Fisher | #A11037 |

Suppliers: CST (Cell Signaling Technologies, Beverly MA, USA), SCBT (Santa Cruz Biotechnology, Santa Cruz, CA, USA), Abcam (Cambridge, UK), Merck Millipore (Billerica, MA, USA), MRC PPU Reagents and Services (Dundee, UK), Sigma-Aldrich (St. Louis, MO, USA).

**Supplementary table 2.** Characteristics of neuroblastoma cell lines

| **Cell line** | **Stage** | ***MYCN* status** | ***ALK* status** | **11q** | ***TP53*** |
| --- | --- | --- | --- | --- | --- |
| CHLA-90 | 4 | Non amplified | F1245V Mut | WT | E286K Mut, NF |
| SK-N-BE(2) | 4 | Amplified | WT | WT | C135F Mut, NF |
| SK-N-AS | 4 | Non amplified | WT | del | Exon 9b AS, NF |
| LA1-5s | 4 | Amplified | F1174L Mut | WT | C182X Mut, NF |
| IMR-32 | 4 | Amplified | PA, WT | WT | WT, functional |
| SH-SY5Y | 4 | Non amplified | F1174L Mut | WT | WT, functional |

PA, partial amplification; WT, wild type; Mut, mutated;AS, alterntative splicing; NF, non-functional.; del, deletion.

**Supplementary table 3.** IC_5_0 values for ABTL0812 and cisplatin in neuroblastoma cell lines

| **Cell line** | **IC_50_ ABTL0812 (μM)** | **IC_50_ Cisplatin (μM)** |
| --- | --- | --- |
| CHLA-90 | 57.87 ± 0.78 | 10.80 ± 1.07 |
| SK-N-BE(2) | 58.70 ± 1.08 | 3.18 ± 1.10 |
| SK-N-AS | 32.49 ± 0.03 | 2.75 ± 1.09 |
| LA1-5s | 50.68 ± 1.34 | 4.79 ± 1.09 |
| IMR-32 | 43.33 ± 0.22 | 0.13 ± 1.02 |
| SH-SY5Y | 43.35 ± 1.40 | 0.20 ± 1.02 |

**Supplementary figure legends**

**Supplementary Figure 1: ABTL0812 is neither mutagenic nor a DNA-damaging agent**. Five *Salmonella typhimurium* strains were incubated with indicated ABTL0812 concentrations (3-5000 µg/plate) without (**a**) or with (**b**) S9 microsomal fraction. Data is presented as the average of three independent experiments ± SD. UT, untreated; Ct, water control. **c** Neuroblastoma cells were treated for 24 h and 48 h with vehicle (Ct), 20 µM ABTL0812 and 25 µM cisplatin. Western blot of the phosphorylated form of histone H2AX was used as indicative of DNA damage. TRIB3 expression was used as an ABTL0812 response biomarker.

**Supplementary Figure 2. *In vivo* toxicity of ABTL0812**. **a** Weight curves of mice treated with vehicle, 120 mg/kg ABTL0812 or 2 mg/kg cisplatin. **b** Complete blood cell count test from mice treated with ABTL0812 or cisplatin. * means out of the reference range.
